# A sclerosing angiomatoid nodular transformation (SANT) mimicking a metachronous splenic metastasis from endometrioid cancer and ovarian cancer

**DOI:** 10.1016/j.ijscr.2019.11.006

**Published:** 2019-11-09

**Authors:** Ryota Koyama, Nozomi Minagawa, Yoshiaki Maeda, Toshiki Shinohara, Tomonori Hamada

**Affiliations:** Department of Gastrointestinal Surgery, Hokkaido Cancer Center, Sapporo, Japan

**Keywords:** Sclerosing angiomatoid nodular transformation, Splenic metastasis, Laparoscopic splenectomy

## Abstract

**Introduction:**

Sclerosing angiomatoid nodular transformation (SANT) of the spleen has been considered a differential diagnosis of splenic lesions since it was originally reported. However, preoperative diagnosis of SANT is often difficult and histopathological examination by surgical resection is required.

**Case presentation:**

Because of a new splenic lesion, a 48-year-old woman was suspected of having metachronous solitary splenic metastasis during her postoperative follow-up for endometrioid and ovarian cancer that occurred 3 years previously. Because there was no metastasis to other sites, laparoscopic splenectomy was successfully performed for diagnosis and treatment. Histopathological examination revealed that the splenic lesion consisted of three distinct splenic vessels, thereby indicating SANT without any cancer cells or lymphoproliferative disorders.

**Conclusion:**

Splenectomy should be considered for the diagnosis and treatment of incidentally detected splenic lesions during follow-up for malignancy.

## Highlights

• The authors present an unusual case of sclerosing angiomatoid nodular transformation (SANT), which newly emerged as a solitary splenic lesion in the postoperative course of endometrioid and ovarian cancer, both of which was resected 3 years ago.
• SANT is often misdiagnosed as malignant lesion and splenectomy is required for its final diagnosis.
• Although preoperative diagnosis is still difficult, SANT must be included into the differential diagnosis of splenic lesion, especially when there is “spoke-wheel pattern” on MRI.

## 1 Introduction

Sclerosing angiomatoid nodular transformation (SANT) is a rare, non-tumorous benign nodular lesion and was initially reported by Martel in 2004 [1]. Because of its radiological resemblance with metastatic lesions, SANT is often resected on suspicion of metastasis from cancers of various origins [[2], [3], [4]]. Its histopathological features include proliferation of various types of splenic vessels, and it can be differentiated from hemangioma owing to its polyclonality [5]. The etiology of SANT is yet to be completely understood. Its diagnosis largely depends on imaging studies; however, currently, no imaging modality is capable of definite diagnosis of SANT. Here we describe the case of a 48-year-old woman who was suspected of having metachronous solitary splenic metastasis from endometrioid and ovarian cancer that was surgically resected approximately 3 years previously. Laparoscopic splenectomy was successfully performed for diagnosis and treatment. We report the features of this case along with a review of the literature. This work has been reported in line with the SCARE criteria [6].

## 2 Presentation of case

**Fig. 1:**
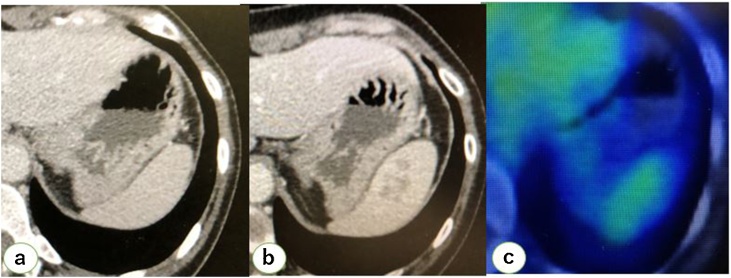
CT and FDG-PET findings. (a–b) CT showing a relatively well-circumscribed, heterogeneously enhanced round mass measuring 25 mm [a) 3 years previously and b) before the present surgery]. (c) FDG-PET showing a slight uptake in the splenic lesion; no other significant findings indicated recurrence at other sites.

**Fig. 2:**
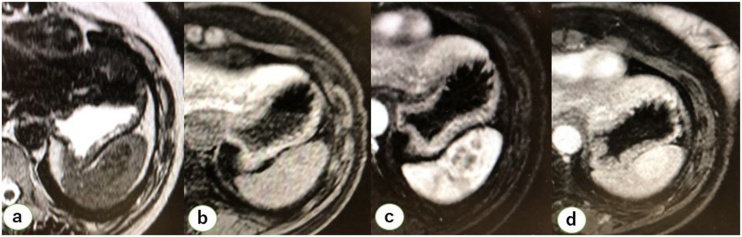
MRI findings. MRI showing a heterogeneous low intensity region within the lesion, indicating fibrosis or hemosiderin, on a T2-weighted image. a) The lesion showed iso-intensity in the spleen, and dynamic study showed gradual enhancement of the lesion [b) 0 s, c) 30 s, d) 180 s] characterized as a “spoke-wheel pattern” on a T1-weighted image.

A 48-year-old woman with a past history of laparoscopic hysterectomy and bilateral salpingo-oophorectomy for endometrioid cancer (pT1aNXM0, pStageⅠA) and left ovarian cancer (endometrioid, pT1aNXM0, pStageⅠA) was diagnosed with a new solitary lesion, which emerged after three years of follow-up, in the spleen. Metachronous solitary splenic metastasis was suspected based on the clinical course. Her past history also included ulcerative colitis (she was administered mesalazine from 19 to 30 years of age and is in complete remission) and thyroid cancer (post-hemithyroidectomy at 42 years of age). She was administered vilanterol trifenatate/fluticasone furoate for cough-variant asthma. On admission, her height was 156 cm and weight was 56 kg; her vital signs were as follows: blood pressure, 119/80 mmHg; heart rate, 74 bpm; oxygen saturation level, 97 % in room air; and body temperature, 36.4 °C. Her mother had brain tumor, which was the only cancer-related family history. Her abdomen was soft and flat with laparoscopic scars and no palpable mass. Her laboratory examination results revealed a marginally elevated free T4 level (1.55 ng/dl); other parameters, including the levels of tumor markers (carcinoembryonic antigen, carbohydrate antigen 19-9, and alpha-fetoprotein) and soluble IL-2 receptor, were within normal limits. Computed tomography revealed no sign of relapse in the pelvis and no swollen lymph nodes. A heterogeneously enhanced round mass measuring 25 mm was detected in the spleen (Fig. 1), and no mass was detected in the thyroid. Magnetic resonance imaging (MRI) revealed iso-intensity in the spleen on T1-weighted images. On T2-weighted images, a heterogeneous low-intensity region was observed within the lesion, indicating fibrosis or hemosiderin. Dynamic study showed gradual enhancement of the lesion (Fig. 2).

**Fig. 3:**
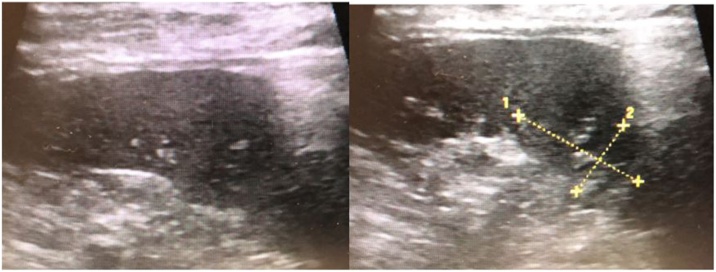
Ultrasonography findings. Ultrasonography revealed microcalcification within the lesion measuring 26 × 16 mm^2^ and calcification along the vessel wall with a poorly marginated lesion.

Ultrasonography revealed microcalcification (26 × 16 mm^2^) along the vessel wall within the lesion; it also revealed that the lesion was poorly marginated (Fig. 3). 18F-2-fluoro-2-deoxyglucose-positron emission tomography revealed a slight uptake in the splenic lesion; otherwise, there were no significant findings indicating recurrence at other sites (Fig. 1). The differential diagnoses included metastatic splenic tumor, SANT, inflammatory pseudotumor (IPT), and splenic abscess.

Laparoscopic splenectomy was successfully performed. Intraoperative findings included the absence of ascites and dissemination; no significant findings were obtained in the pelvis. The spleen was resected by clipping the splenic vein and artery and was extracted from the abdominal cavity via the umbilicus with a minor extension of the incision.

**Fig. 4:**
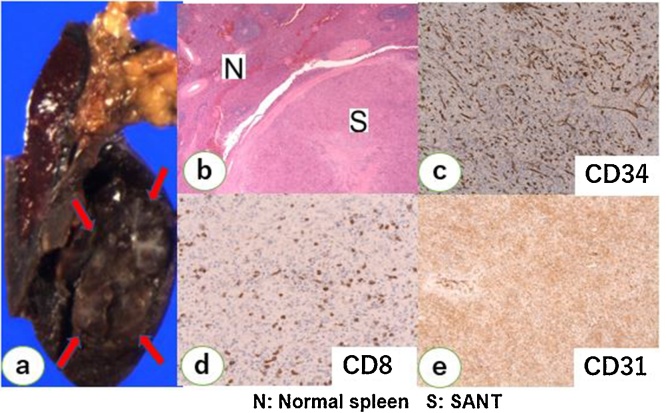
Pathological findings. (a) Macroscopically, a dark-reddish nodular lesion measuring 23 × 20 × 15 mm^3^ with a color similar to that of the background spleen was observed. b) Histopathological examination showing a well-circumscribed lesion mainly composed of a red pulp-like structure with irregular fibrosis in the stroma. c–e) Immunohistochemistry showing coexistence of three types of vessels (capillary of CD34+/CD8−/CD31+, splenic sinusoid-like vessels of CD34+/CD8−/CD31+, and small vein of CD34+/CD8−/CD31+).

The resected specimen had a dark-reddish nodular lesion measuring 23 × 20 × 15 mm^3^ (Fig. 4). The color was similar to that of the background spleen and was marginally unclear. The histopathological examination revealed a well-circumscribed lesion mainly comprising a red pulp-like structure with irregular fibrosis in the stroma (Fig. 4). Immunohistochemistry revealed the coexistence of three types of vessels (capillary of CD34+/CD8−/CD31+, splenic sinusoid-like vessels of CD34+/CD8−/CD31+, and small vein of CD34+/CD8−/CD31+) (Fig. 4). There was no sign of cancer cells or lymphoproliferative disorders, including malignant lymphoma.

The postoperative course was uneventful, and the patient was discharged on the 12th day postoperatively. Currently, the patient receives pneumococcal vaccine and no additional treatment.

## 3 Discussion

SANT, a rare splenic tumor, was initially reported by Martel in 2004 following the examination of a 25-mm mass-forming lesion with a unique finding [1]. Most cases of SANT are detected incidentally upon radiographic examination of asymptomatic patients. Most patients exhibit no symptoms; however, some experience abdominal pain or discomfort [7]. No splenic ruptures due to SANT have been reported. There is an increase in the rate of incidentally detected cases, such as those identified during follow-up after surgery for malignant lesions or during routine medical check-up. In the postoperative follow-up course, new lesions need to be differentiated from metastasis. Because of its ability to grow its size, SANT is often mistaken for metastasis and is surgically resected. Suspected malignancy includes uterine clear cell carcinoma [2], colon cancer [3], and rectal cancer [4]. Although SANTs can be mistakenly resected, they should be treated as malignant lesions until the final diagnosis is achieved. To date, there has been no report on specific laboratory data on factors, including biomarkers. Preoperative diagnosis is mainly determined radiographically. Karaosmanoglu described a “spoke-wheel pattern” that characterizes SANT in the spleen. Centripetal filling in a radiating pattern is observed on dynamic MRI; this was also observed in the present study, and this finding may be useful but not definitive (Fig. 2) [8]. Image-guided percutaneous procedures have been reported but are generally not performed owing to complications such as hemorrhage and dissemination [9]. Therefore, the diagnosis of focal splenic lesions chiefly depends on surgical resection of the spleen. Lately, splenectomy is being performed laparoscopically; however, surgeons must be careful not to damage the spleen unless it is proven malignant. If malignancy is highly suspected, surgeons should not hesitate to switch to laparotomy.

Pathological examination of the splenic lesion is used to achieve the final diagnosis of SANT. Macroscopically, SANT is a well-circumscribed solitary mass with heterogeneous angiomatous lesions forming in the splenic nodules [1]. Histopathologically, SANT is characterized by nodular lesions composed of three types of vessels: capillaries [CD34(+)/CD8(−)/CD31(+)], sinusoid-like vessels [CD34(−)/CD8(+)/CD31(+)], and small veins [CD34(−)/CD8(−)/CD31(+)] [1]. Cavernous hemangioma is the most frequent vascular lesion occurring in the spleen. Furthermore, littoral cell angioma, which originates from splenic sinus lining cells, is a vascular lesion in the spleen. However, these lesions are different from SANT because they are composed of monophasic vascular structures and exhibit scarce fibrosis. The exact etiology of SANT is still unknown. One hypothesis states that it is caused by blockage of the vascular outflow in the red pulp, leading to vascular proliferation and CD8 downregulation in the sinusoid endothelium as well as stromal fibrosis [1]. Another hypothesis states that SANT is a hamartomatous lesion originating from the red pulp and that it interacts with the noncancerous proliferating stroma [10]. Recently, based on genetic analysis of human androgen receptor alpha (*HUMARA*), Chang reported that SANT is essentially a non-tumorous reactive lesion [5]. IPT is a nodular lesion with invasion of nonspecific inflammatory cells and regenerative reaction of mesenchymal cells, and its etiology is similar to that of SANT. The difference between SANT and IPT is still unclear, and further investigation is warranted [11]. SANT is a non-neoplastic benign lesion and demonstrates good prognosis after surgical resection of the spleen. Further information about the etiology of SANT is required to make its diagnosis less invasive.

## 4 Conclusion

When a splenic lesion is incidentally detected, SANT should be considered as a differential diagnosis. If the lesion is related to a malignancy, splenectomy should be considered for both diagnosis and treatment. However, when it is not linked to any malignancy, careful observation could be an option.

## Declaration of Competing Interest

The authors (RK, NM, YM, TS & TH) declare no conflicts of interests or disclosures.

## Funding

This work received no funding.

## Ethical approval

This study is exempt from ethical approval in our institution.

## Consent

Written informed consent was obtained from the patient for publication of this case report and accompanying images. A copy of the written consent is available for review by the Editor-in-Chief of this journal on request.

## Author contribution

RK is the primary investigator and contributed to conceptualization, data collection and drafting the manuscript. NM, YM, TS, TH supervised and checked the manuscript. All authors have read and approved this manuscript for publication.

## Registration of research studies

The name of the registry is research registry. The unique identifying number (UIN) is researchregistry5202.

## Guarantor

Ryota Koyama.

Tomonori Hamada.

## Provenance and peer review

Not commissioned, externally peer-reviewed.
